# Evolving surgical management of pediatric vesicoureteral reflux: is open ureteral reimplantation still the ‘Gold Standard’?

**DOI:** 10.1590/S1677-5538.IBJU.2020.99.05

**Published:** 2020-02-20

**Authors:** Andrew J. Kirsch, Angela M. Arlen

**Affiliations:** 1 Emory University School of Medicine Children's Healthcare of Atlanta AtlantaGA USA Emory University School of Medicine Children's Healthcare of Atlanta, Atlanta, GA 30328, USA; 2 Yale University School of Medicine Department of Urology New HavenCT USA Department of Urology, Yale University School of Medicine, New Haven, CT 06520, USA

**Keywords:** Vesico-Ureteral Reflux, Ureteral Diseases, Pediatrics

## Abstract

Vesicoureteral reflux, the retrograde flow of urine from the bladder into the upper urinary tract, is one of the most common urologic diagnoses in the pediatric population. Once detected, therapeutic options for urinary reflux are diverse, ranging from observation with or without continuous low-dose prophylactic antibiotics to a variety of operative interventions. While a standardized algorithm is lacking, it is generally accepted that management be tailored to individual patients based on various factors including age, likelihood of spontaneous resolution, risk of subsequent urinary tract infections with renal parenchymal injury, and parental preference. Anti-reflux surgery may be necessary in children with persistent reflux, renal scarring or recurrent pyelonephritis after optimization of bladder and bowel habits. Open, laparoscopic/robot-assisted and endoscopic approaches are all successful in correcting reflux and have been shown to reduce the incidence of febrile urinary tract infections.

## INTRODUCTION

Vesicoureteral reflux (VUR) is one of the most common urologic diagnoses affecting children, with an estimated prevalence of 0.4-1.8% in the general pediatric population, 10-20% of those with antenatal hydronephrosis, and up to 40% of children with a history of febrile urinary tract infection (UTI) (1-3). Moreover, newborns have a higher propensity for renal injury and are at higher risk of having VUR after initial febrile UTI (4). Management options for urinary reflux encompass a broad spectrum, ranging from observation with or without continuous low-dose antibiotic prophylaxis to a variety of operative interventions. In recent years, aggressive reflux management has been called into question and a more selective approach to the diagnosis and treatment of VUR has gained favor, with an emphasis on identifying children at risk for recurrent pyelonephritis and renal scarring (5, 6).

Successful surgical correction of VUR in children can be achieved via open, laparoscopic or robot-assisted laparoscopic or endoscopic approaches and fortunately for pediatric urologists and surgeons, all are potentially successful and have their merits. The decision *how to best* surgically manage primary VUR is dependent on a multitude of factors, including the influence of training and personal experience of the surgeon, and the impact of published literature. Biases exist in data reporting and selective data use, as well as potential economic benefits to the surgeon using one approach over another. Ideally, after careful consideration of the various pros and cons of each approach, shared decision-making between the family and the surgeon will lead to the most appropriate intervention for a given patient.

Open ureteral reimplantation (OUR), robot-assisted laparoscopic extravesical reimplantation (RALUR), and endoscopic injection (EI) have all proven effective at correcting VUR and preventing febrile urinary tract infections (7, 8). Defining “success” postoperatively is key to comparing the outcomes of each surgical procedure and analyzing available literature. This review will emphasize how implementation of an individualized care model, taking into consideration current data on the benefits and complications of anti-reflux surgery, is leading to the emergence of new “gold standards” in the surgical management of VUR. Today, the “gold standard” surgical approach must result in a reduction of febrile UTIs, have low morbidity and be reproducible, while also being acceptable to parents of children with VUR.

### How is “Success” of Anti-Reflux Surgery Defined?

Management goals of VUR include prevention of recurrent pyelonephritis and renal injury while minimizing the morbidity of associated treatment and follow-up (9). Surgical success can be defined both radiographically (i.e. no VUR on postoperative voiding cystogram) and clinically (i.e. no postoperative febrile UTIs) (10). Arguably the prevention of recurrent febrile UTIs, the very reason for obtaining a VCUG and diagnosing VUR in the first place, should be considered the *primary* definition of success and thus more important in the long term than radiographic findings. The clinical definition of success also underscores the importance of screening for and treatment of bladder-bowel dysfunction (BBD) prior to any anti-reflux procedure, as dysfunctional elimination influences not only surgical success but the risk of febrile UTI (9).

### Open Ureteral Reimplantation

Creation of a ureteroneocystostomy is an elegant surgical skill that has helped to define the field of pediatric urology for over 50 years, and various open reimplantation techniques have been described including both intravesical and extravesical approaches. The Cohen cross-trigonal reimplantation is the most widely utilized intravesical ureteroneocystostomy technique, due to reliable results and broad applicability. It maintains the same ureteral hiatus in the bladder wall, with the ureter advanced through a submucosal tunnel across the trigone to the contralateral bladder wall (11-13). It is well-suited for small or thick-walled bladders, as ureteral advancement across the back wall of the bladder rarely results in kinking or obstruction. The technique utilized for extravesical ureteral reimplantation is the Lich-Gregoir or one of its modifications. In this approach, the juxtavesical ureter is dissected free but not detached and a detrusor trough is created by incising the serosa and detrusor down to the mucosa, extending laterally from the ureteral hiatus. The refluxing ureter is placed into the trough, and the detrusor is closed over the ureter, creating a flap valve mechanism without opening the bladder (14, 15).

The idiom “tried-and-true” describes OUR perfectly as it has long been touted as the “gold standard” with radiographic success rates reported to be up to 98% for grades I-IV. Given this high success rate, the need for a routine postoperative VCUG is usually dictated by the patient's postoperative clinical course and is not routinely recommended (9). Despite being regarded as the gold standard, there is surprisingly limited recent literature describing the long-term clinical outcomes after open reimplantation. The International Reflux Study in Children reported a 5-year UTI incidence of 39% following surgery for dilating reflux (grade III-V), while clinical pyelonephritis occurred in 10% (16). In 2013, Nelson et al. published a large series of over 1000 children undergoing OUR, with radiographic success achieved in 93.5% (96.5% in those without ureteral tailoring). During a median follow-up of 2.9 years, 6.5% of children developed clinical pyelonephritis while the incidence of any postoperative UTI was 21.8% (17). This underscores the need to counsel caregivers that while OUR is successful at correcting VUR and therefore preventing pyelonephritis, postoperative UTI remains relatively common. As anticipated, failure was higher in girls, those with renal scarring, higher VUR grade, and in those with increased number of preoperative UTIs. Furthermore, the morbidity as measured by emergency room visits and hospitalizations postoperatively is notably higher when OUR is compared to EI (18). These findings underscore the reality that OUR may not be superior to either EI or RALUR with regards to clinical outcomes rather, it is one of several surgical options for correcting primary VUR.

### Robot-Assisted Laparoscopic Ureteral Reimplantation

Use of minimally invasive surgical techniques has become increasingly common in the pediatric population over the past decade, and robotic technology has served to bridge the gap between open and laparoscopic surgery with magnified three-dimensionality and superior stereoscopic visualization (19, 20). Given the need for delicate intracorporeal suturing, robotic surgery is particularly advantageous for reconstructive procedures (21, 22). Robotic reimplantation is typically performed via an extravesical approach and has gained increasing acceptance (19, 23).

VUR resolution rates after extravesical robotic ureteral reimplantation reported in the literature range from 66.7 to 100% in multiple relatively small series; the overall radiographic success rate upon pooling these series is 91% (24). A multi-institutional retrospective study reported radiographic success of 87.9%; Clavien grade III and lower complications were seen in fewer than 10%, including 3.9% of cases with transient acute urinary retention after bilateral RALUR, a known complication of bilateral extravesical reimplantation (25). The same consortium of robotic surgeons then conducted a prospective multicenter study on RALUR, and reported a slightly higher resolution rate of 93.8% with a 91.9% clinical success rate (8.1% incidence of postoperative febrile UTI) (26).

In comparison to OUR, RALUR has been associated with decreased morbidity, less postoperative pain, lower analgesic requirements, quicker postoperative recovery, and shorter hospital stays. However, there are multiple reports of higher complication rates with the robotic compared to the open approach and while success rates approach that of OUR (27, 28), shared decision making with caregivers helps determine the best approach for an individual child. As with other robot-assisted laparoscopic operations, advantages compared to an open approach seem most apparent in older children and must be balanced against operative time and cost considerations. Furthermore, evidence suggests that a hidden Pfannenstiel incision may be more desirable than visible port sites used in the robotic approach (29). While multi-institutional studies support RALUR as a safe and effective treatment option in older patients when performed by experienced surgeons (25, 26), efforts to identify patient and technique factors associated with optimal surgical outcomes while minimizing complications remains key.

### Endoscopic Injection

Endoscopic correction using an injectable bulking agent as an alternative to open anti-reflux surgery was initially described nearly four decades ago. O'Donnell and Puri popularized the concept by performing subureteric injections using Teflon paste, i.e the “STING” (subureteric teflon injection) procedure (30). Double hydrodistention implantation technique (Double HIT), the hallmark of which is ureteral hydrodistention, allows for direct visualization and injection into the intraluminal ureteral submucosal plane and improved success rates (31). In the Double HIT method, the needle is placed into the distended ureteral orifice and inserted in the mid-ureteral tunnel at the 6 o'clock position (rather than below the orifice as with the STING technique). Dx/HA is injected until a sufficient bulge is produced, coapting the detrusor tunnel. The second injection at the distal most aspect of the intravesical ureteral tunnel results in coaptation of the ureteral orifice. Hydrodistention, with the bladder nearly empty, is performed following each injection to monitor progress and ensure adequate ureteral coaptation (32).

Proponents of the endoscopic approach tout benefits including the ambulatory nature and decreased patient morbidity, while opponents note both higher initial radiographic failure and recurrence rates compared to ureteral reimplantation. Success rates of up to 94% have been reported by our group with the Double HIT methods (10, 33, 34). Other studies using varying techniques and injected volumes, have demonstrated wide variability with reported treatment failure rates of 6-50%; outcomes are dependent upon the technique utilized, injected material, VUR grade and surgeon experience (35). Aggregate literature suggests that endoscopic therapy is relatively effective for the treatment of most primary VUR, while stressing the importance of reflux grade and structural/functional bladder anomalies on ultimate success rates. In a systematic meta-analysis evaluating Dx/HA for pediatric VUR, the estimated overall reported success rate for endoscopic therapy was 72% with 89% success for grade I, 83% for grade II, 71% for grade III, 59% for IV and 62% for grade V reflux (36). It is important to re-emphasize that this meta-analysis included various injection methods, volumes of injected material, and surgeon experience. Despite the potential for lower radiographic success rates, families and surgeons alike are drawn EI, due to the minimally invasive nature and similar clinical success. In some studies using the STING technique and relatively lower volume of injection, length of follow-up has had an impact on EI success rates. Radiographic recurrence of reflux after initial successful STING injection appears to be around 15-20% within several years and is stable thereafter (37-39). Late radiographic failures are hypothesized to be secondary to the biodegradable nature of Dx/HA; the clinical significance of late recurrent VUR in the absence of symptomatic infections is unclear, however, down-grading VUR may play an important role.

Our experience using Dx/HA over nearly twenty years has been quite good with outcomes similar to that reported for OUR and RALUR. In a series of 229 children undergoing EI with Dx/HA, 14 patients (6.3%) experienced a postoperative febrile UTI during mean clinical follow-up of 34.7 months (33). In a longer-term study with greater than 5-year (median 8.4 year) follow-up, a 10.2% incidence of postoperative febrile UTI was reported (40). These studies underscore the long-term clinical success rate of Double HIT for primary VUR. We no longer suggest VCUGs following EI since studies have confirmed no benefit to those patients who have undergone a postop VCUG compared to those who have not (33).

The biodegradable nature of the Dx/HA copolymer and its role in long-term failures prompted development of the synthetic, non-biodegradable Polyacrylate Polyalcohol Copolymer (PPC, Vantris^®^) (41). PPC has had promising short and long-term results outside of the United States since its introduction in 2010 (42). In a comparative study, Warchol and colleagues reported considerably higher success rates after a single injection with PPC compared to Dx/HA (43). These findings were confirmed in a recently published study, which reported a PPC radiographic success rate of 92.2% compared to 75.7% for Dx/HA, controlling for grade and injection technique (44). Studies have indicated a higher complication rate, notably ureteral obstruction, using PPC. As a result, most agree that the Double HIT method should not be used with PPC.

### What Do Patients and Parents Prefer?

**Figure 1:**
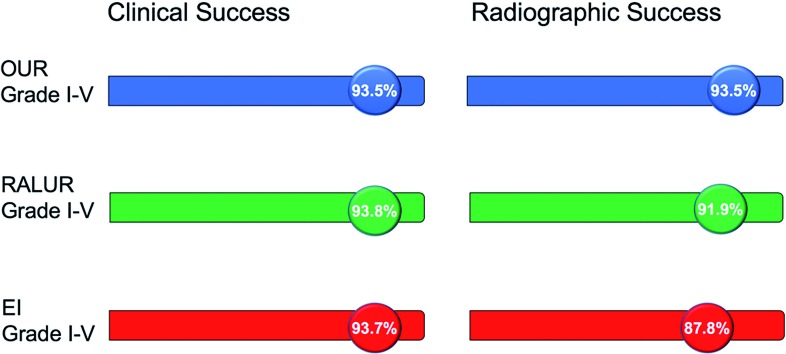
Comparison of clinical and radiographic success of open ureteral reimplantation (14), robotic ureteral reimplantation (26) and Dx/HA injection (33).

Shared decision making, the collaborative process of clinicians and patients (or parental surrogates) making medical decisions together, takes into consideration not only risks and benefits of a given intervention, but also the preferences, goals, and concerns of the family before arriving at a decision (45). Perhaps nowhere is this concept more relevant in pediatric urology than in diagnoses of primary VUR, where ‘optimal’ treatment remains heavily debated and a universal management algorithm is lacking. Furthermore, the clinical success rates of OUR, RALUR and EI are all similar, underscoring the merits of each approach and the need for individualized care (Figure-1). In summary, what may be ideal for one child may not be the “gold standard” for another.

In 2011, the American Academy of Pediatrics (AAP) revised the practice parameters regarding diagnosis and management of initial febrile UTIs in infants and young children aged 2 to 24 months; guidelines now recommend that children with initial febrile UTI undergo a renal-bladder ultrasound, but forego VCUG unless indicated by sonographic findings (i.e. hydronephrosis, scarring) (5). The revised guidelines challenge the utility of aggressive diagnosis and subsequent management of *all* primary reflux, representing a shift towards a more selective approach. Not surprisingly, national trends in the surgical management of primary reflux in children have revealed significant declines in OUR and EI since the publication of the revised AAP UTI guidelines (46). However, OUR has been on a statistically significant decline well before guideline publication, showing a downward trend since before 2004. Accounting for this shift was the emergence of EI in the USA in 2001. Since 2008, RALUR has shown a modest rise in utilization further competing with OUR. It is difficult to discern the role that parental preference plays in these trends, but the decline of open surgical repairs, may suggest that families and clinicians are opting for more minimally invasive options.

## CONCLUSIONS

**Figure 2:**
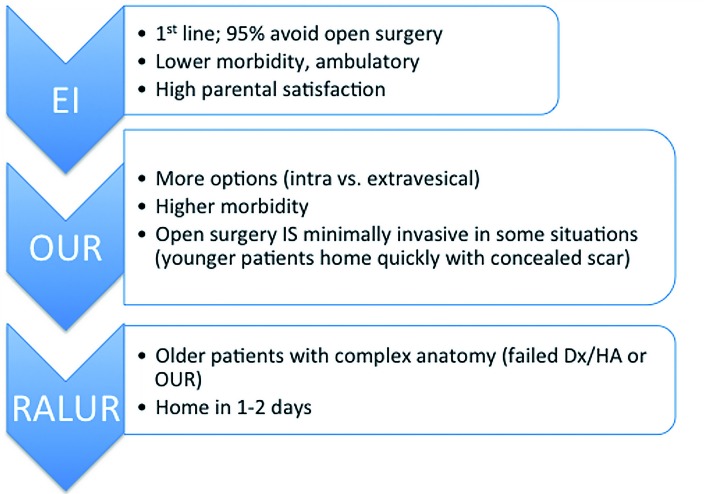
EI may be considered first line surgical therapy for most cases of grade II-IV VUR, owing to its ambulatory nature and good clinical success. OUR offers more approaches with high radiographic and clinical success rates, but higher morbidity compared to EI must be considered. RALUR is an option in older children but is typically limited to an extravesical approach.

Over the past decade, there has been a shift towards a more selective approach to surgical management of primary urinary reflux, aimed at identifying children most likely to experience the untoward effects of recurrent pyelonephritis who would therefore benefit from surgical repair. While open ureteroneocystostomy, robot-assisted laparoscopic reimplantation, and endoscopic injection have differing ranges of reported radiographic success, it is important to note their rates of clinical success are similar. If the ultimate goal is prevention of febrile urinary tract infections, we must also acknowledge a shift in what is considered the “gold standard” in operative management of VUR. Based on our experience and that reported by others, we include our surgical treatment algorithm (Figure-2), emphasizing that there are several “gold standards” from which to optimize the care of an individual child with primary VUR.
